# Genomic and phenotypic characterization of *Streptococcus mutans* isolates suggests key gene clusters in regulating its interaction with *Streptococcus gordonii*

**DOI:** 10.3389/fmicb.2022.945108

**Published:** 2022-08-12

**Authors:** Shanshan Liu, Yu Sun, Yudong Liu, Fuyong Hu, Li Xu, Qingwei Zheng, Qinglong Wang, Guojin Zeng, Kai Zhang

**Affiliations:** ^1^Department of Stomatology, The First Affiliated Hospital of Bengbu Medical College, Bengbu, China; ^2^Department of Stomatology, Bengbu Medical College, Bengbu, China; ^3^Department of Biochemistry and Molecular Biology, Bengbu Medical College, Bengbu, China; ^4^Department of Histology and Embryology, Bengbu Medical College, Bengbu, China; ^5^Department of Epidemiology and Health Statistics, Bengbu Medical College, Bengbu, China; ^6^Anhui Key Laboratory of Infection and Immunity, Bengbu Medical College, Bengbu, China

**Keywords:** genetic analysis, clinical isolates, dental caries, *Streptococcus mutans*, *Streptococcus gordonii*

## Abstract

*Streptococcus mutans* (*S. mutans*) is one of the primary pathogens responsible for dental caries. *Streptococcus gordonii* (*S. gordonii*) is one of the early colonizers of dental plaque and can compete with *S. mutans* for growth. In the present analysis, we explored key target genes against *S. gordonii* in *S. mutans* using 80 *S. mutans* clinical isolates with varying capabilities against *S. gordonii*. A principal coordinate analysis revealed significant genetic diversity differences between antagonistic and non-antagonistic groups. Genomic comparisons revealed 33 and 61 genes that were, respectively, positively and negatively correlated with *S. mutans* against *S. gordonii*, with RNA-sequencing (RNA-seq) highlighting 11 and 43 genes that were, respectively, upregulated and downregulated in the antagonistic group. Through a combination of these results and antiSMASH analysis, we selected 16 genes for qRT-PCR validation in which the expression levels of SMU_137 (malate dehydrogenase, mleS), SMU_138 (malate permease, mleP), SMU_139 (oxalate decarboxylase, oxdC), and SMU_140 (glutathione reductase) were consistent with RNA-seq results. SMU_1315c-1317c (SMU_1315c transport-related gene) and SMU_1908c-1909c were, respectively, downregulated and upregulated in the antagonistic group. The expression patterns of adjacent genes were closely related, with correlation coefficient values greater than 0.9. These data reveal new targets (SMU_137–140, SMU_1315c-1317c, and SMU_1908c-1909c) for investigating the critical gene clusters against *S. gordonii* in *S. mutans* clinical isolates.

## Introduction

Dental caries is a highly prevalent infectious bacterial disease, ranking first in prevalence among 328 diseases worldwide in 2016 (Vos et al., 2017). Dental caries can adversely impact the quality of life of affected patients, resulting in complications including pain and, in severe cases, tooth loss (Aldosari et al., 2021; Amarasena et al., 2021; Santos et al., 2021). *Streptococcus mutans* (*S. mutans*) is an essential cariogenic bacterium that metabolizes carbohydrates within the oral cavity and thereby lowers the local pH at the tooth surface (Loesche, 1986; OmerOglou et al., 2022). When this pH value drops to 5.5 or below, it can result in tooth surface demineralization and dental caries development (He et al., 2022).

*Streptococcus gordonii* (*S. gordonii*) is a Gram-positive bacterium included among some of the initial colonizers of the dental plaque biofilm (Pearce et al., 1995). The metabolic production of hydrogen peroxide produced by *S. gordonii* can inhibit the growth of *S. mutans*, and the production of alkaline ammonia can mitigate the localized acidity on the tooth surface, thereby helping to suppress cariogenesis (Liu and Burne, 2009; Cheng et al., 2020; Liu et al., 2022). Studies also showed that the detection rate of *S. mutans* in dental plaque is positively correlated with dental caries, while the detection rate of *S. gordonii* is negatively associated with dental caries (Saraithong et al., 2015; Tao et al., 2015). The activity of *S. mutans* to antagonize *S. gordonii* and colonize the tooth surface is thus a prerequisite for its cariogenic activity.

*S. gordonii*, *Streptococcus sanguinis* and other pioneer bacteria are effectively suppressed by mutacin IV, a bacteriocin released by *S. mutans* (Merritt et al., 2016). The comC gene encodes competence-stimulating peptide (CSP). ComC is processed and secreted by an ABC transporter nlmTE (SMU_286/SMU_287, previously designated comAB) to generate a 21-residue peptide (CSP-21; Hale et al., 2005). The SepM is a 346 amino acid cell wall anchor protein encoded by the SMU_518 consisting of transmembrane, PDZ, and C-terminal regions (amino acids 10–26, 131–195, and 233–314, respectively; Hossain and Biswas, 2012a; Biswas et al., 2016; Bikash et al., 2018). The CSP-21 can be cleaved to the 18-amino acid CSP-18 by SepM such that it can then interact with the ComDE two-component regulatory system, resulting in the histidine kinase membrane-bound ComD receptor autophosphorylates, and the phosphate is transferred to the cognate cytoplasmic response regulator ComE; once phosphorylated ComE binds to the promoter region of the genes that encode mutacin IV (nlmA and nlmB), thereby upregulating this bacteriocin (Hossain and Biswas, 2012a).

In addition to the genes associated with the production of mutacin IV, it is unknown if other genes play a crucial role in the virulence of *S. mutans* against *S. gordonii*. The significant promoting role of *S. mutans* in the onset of dental caries, the role of *S. gordonii* in inhibiting the growth of *S. mutans* and buffering the local pH value of tooth surface demonstrated that the antagonism of *S. mutans* against the growth of *S. gordonii* is of significance for the prevention and treatment of dental caries. In this study, we evaluated the effect of 80 *S. mutans* clinical isolates on *S. gordonii* antagonism and divided them into antagonisticand non-antagonistic groups, and analyzed whether there were differences between the two groups in the genome through phylogenetic tree and principal component analyses. On this basis, we speculated that differentially distributed genes and differentially expressed genes between the two groups may play an important role in the growth of *S. mutans* against *S. gordonii*. We then carried out the following analysis: (1) we found the core genes and differentially distributed genes (DDGs) between the two groups and analyzed the function of these genes; (2) mutacin is a secondary metabolite produced by *S. mutans* and plays an important role in bacterial interactions. We searched for genes involved in secondary metabolites in *S. mutans* through antiSMASH bioinformatics analysis; (3) we searched for differentially expressed genes by RNA sequencing of isolates between the antagonistic group and the non-antagonistic group; (4) we analyzed the common genes shared in 1–3 and these genes were the key candidates participated in the regulation of the growth of *S. mutans* against *S. gordonii*; (5) we used qRT-PCR to validate the role of these key genes in *S. mutans* antagonizing *S. gordonii* using *S. mutans* clinical isolates.

## Materials and methods

### Origin of *Streptococcus mutans* clinical isolates

**Figure 1:**
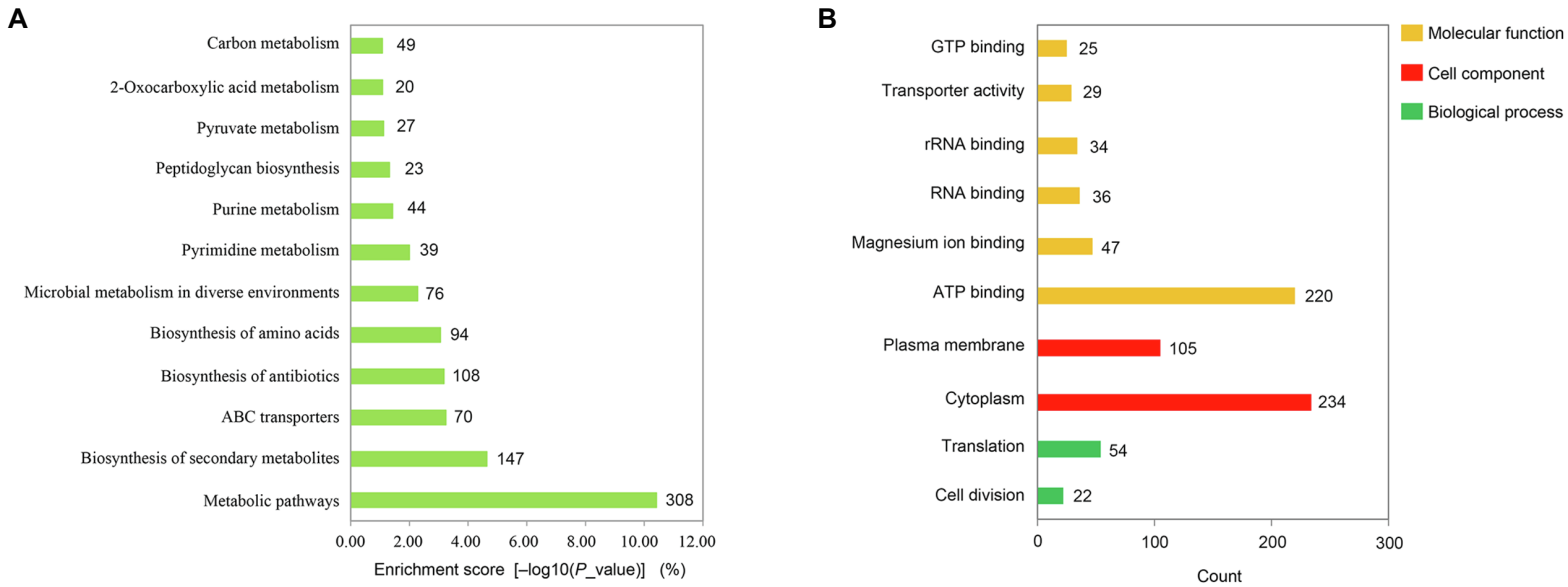
Core gene functional analysis. **(A)** KEGG pathways enriched for core genes. The pathways were sorted based on the enrichment score. **(B)** GO terms enriched for core genes. Each part’s GO terms (molecular function, cell component, or biological process) were ordered by the gene number proportion.

**Figure 2:**
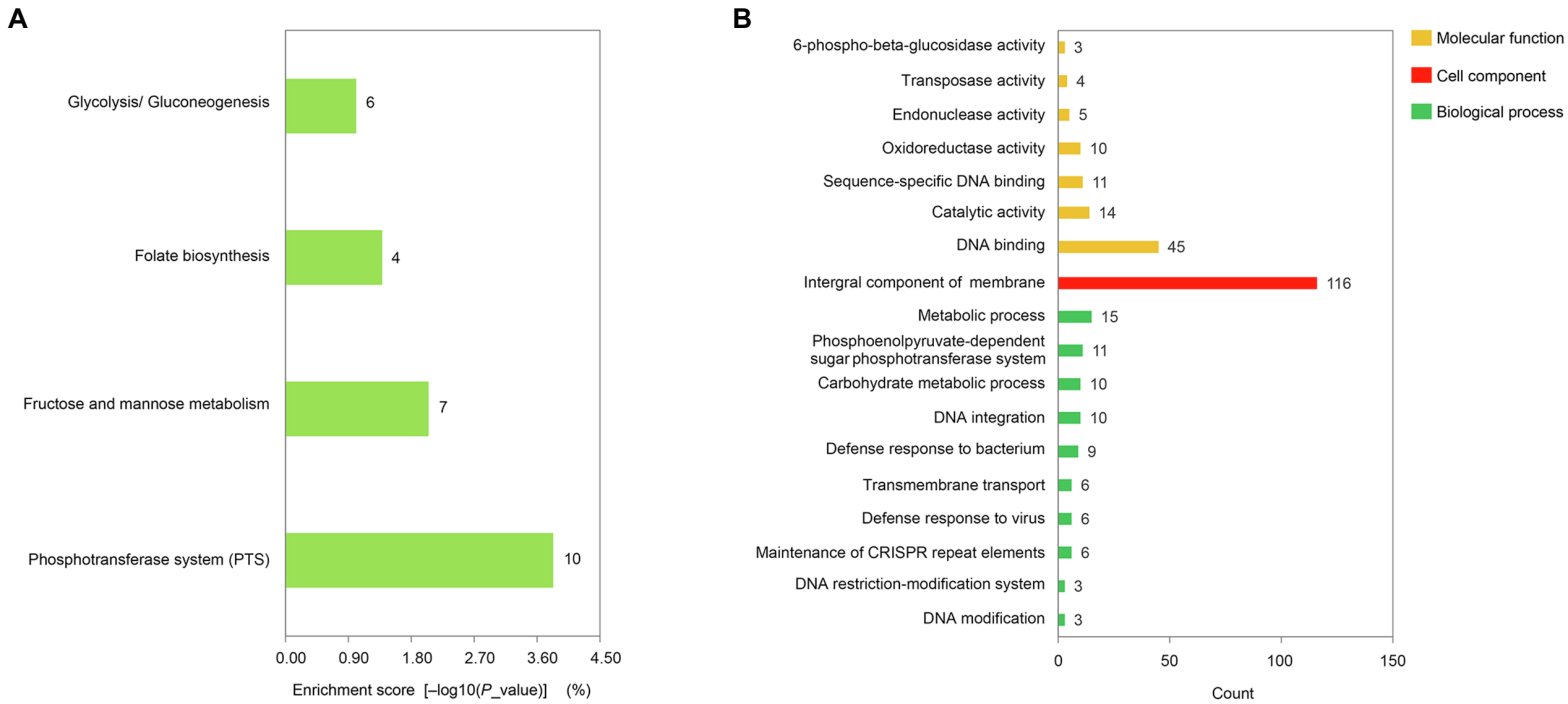
Functional analysis of non-core genes. **(A)** KEGG pathways enriched for non-core genes. **(B)** GO terms enriched for non-core genes. The categories were sorted in the same order as shown in Figure 1.

80 *S. mutans* isolates used in this study were preserved in our laboratory. In our previous study, we reported the source, including dental caries status and the number of kindergartens in these samples (Figures 1, 2; Liu et al., 2020). Briefly, 62 (SMB1-SMB62) and 18 (SMB63-SMB80) isolates were collected from children with caries and caries-free, respectively. These children were from three kindergartens in Bengbu city, Anhui Province, China. The First Affiliated Hospital of Bengbu Medical College provided ethical approval ([2017] KY011) for the present study.

### Average nucleotide identity analysis

Average nucleotide identity (ANI) values for 80 *S. mutans* clinical isolates, *S. mutans* UA159, and 40 other species downloaded from National Center for Biotechnology Information (NCBI; 38 Streptococci sp., *Fusobacterium periodonticum*, and *Neisseria sicca*; Table 1) were calculated as reported previously by Goris et al. (2007). Briefly, genomic sequences from one ‘query’ genome in the genomic pair being compared were cut into 1,020 nucleotide (nt) fragments that were then searched against the whole genome sequence of the other ‘reference’ genome within the pair using BLAST 2.2.26. The mean identity of all BLASTN matches exhibiting >30% overall sequence identity over an alignable region of at least 70% of the sequence length was calculated as the ANI between the query and reference genomes.

**Table 1:** The genome data for the 40 additional species included in average nucleotide identity analyses.

ID	Species	Genome
1	*Streptococcus sinensis*	GCA_000767835.1
2	*Fusobacterium periodonticum* 2_1_31	GCA_003019755.1
3	*Streptococcus constellatus* 925_SCON	GCA_001075725.1
4	*Streptococcus parasanguinis* ATCC 15912	GCA_000164675.2
5	*Neisseria sicca* ATCC 29256	GCA_000174655.1
6	*Streptococcus ratti* ATCC 31377	GCA_008803015.1
7	*Streptococcus peroris* ATCC 700780	GCA_000187585.1
8	*Streptococcus infantis* DD18	GCA_001579645.1
9	*Streptococcus pseudopneumoniae* IS7493	GCA_000221985.1
10	*Streptococcus parauberis* KCTC 11537	GCA_000213825.1
11	*Streptococcus anginosus* NCTC 10713	GCA_900636475.1
12	*Streptococcus downei* MFe28 NCTC 11391	GCA_900459175.1
13	*Streptococcus australis* NCTC 13166	GCA_900476055.1
14	*Streptococcus suis* NCTC10234	GCA_900475585.1
15	*Streptococcus intermedius* NCTC11324	GCA_900475975.1
16	*Streptococcus constellatus* NCTC11325	GCA_900459125.1
17	*Streptococcus oralis* ATCC 35037 NCTC11427	GCA_900637025.1
18	*Streptococcus vestibularis* NCTC12167	GCA_900636445.1
19	*Streptococcus canis* NCTC12191	GCA_900636575.1
20	*Streptococcus sobrinus* NCTC12279	GCA_900475395.1
21	*Streptococcus thermophilus* NCTC12958	GCA_900474985.1
22	*Streptococcus infantarius* NCTC13760	GCA_900459445.1
23	*Streptococcus massiliensis* NCTC13765	GCA_900459365.1
24	*Streptococcus urinalis* NCTC13766	GCA_900636885.1
25	*Streptococcus gallolyticus* NCTC13773	GCA_900475715.1
26	*Streptococcus pseudoporcinus* NCTC13786	GCA_900637075.1
27	*Streptococcus cristatus* NCTC13807	GCA_900478185.1
28	*Streptococcus uberis* NCTC3858	GCA_900475595.1
29	*Streptococcus dysgalactiae* NCTC6181	GCA_900636815.1
30	*Streptococcus pneumoniae* NCTC7465	GCA_001457635.1
31	*Streptococcus sanguinis* NCTC7863	GCA_900475505.1
32	*Streptococcus gordonii* NCTC7865	GCA_900475015.1
33	*Streptococcus equinus* NCTC8140	GCA_900636465.1
34	*Streptococcus pyogenes* NCTC8198	GCA_002055535.1
35	*Streptococcus salivarius* NCTC8618	GCA_900636435.1
36	*Streptococcus equi* NCTC9682	GCA_900637675.1
37	*Streptococcus agalactiae* SA111	GCA_900078265.1
38	*Streptococcus mitis* SK637	GCA_000722765.2
39	*Streptococcus porci* DSM 23759	GCA_000423765.1
40	*Streptococcus iniae* YSFST01-82	GCA_000831485.1

### The activity of *Streptococcus mutans* against *Streptococcus gordonii*

We used a bacteriocin assay to evaluate the activity of *S. mutans* against *S. gordonii* (Huang et al., 2018; Liu et al., 2019). The positive reference strain was *S. mutans* UA159.Each isolate was incubated overnight in brain heart infusion (BHI) broth at 37°C under 5% CO_2._ Ten microliters of each *S. mutans* clinical isolate with an OD600 of 0.3 were added to BHI agar. We inoculated equal amounts of *S. gordonii* (ATCC 10558) adjacent to the *S. mutans* sample after 12 h of incubation. The agar plate medium was then left to culture for another 12 h. Finally, S gordonii clearing zones represent *S. mutans*’s activity against *S. gordonii*. Three biological replicates are used in the investigation. According to the activity of *S. mutans* against *S. gordonii*, we divided the 80 *S. mutans* into the antagonistic group and the non-antagonistic group.

### Genetic diversity estimation

Genomic distances among the genomes of these isolates were determined using MASH^1^ (Ondov et al., 2016, 2019). Analysis (PCoA) was conducted using the R “vegan::pcoa” package. Wilcoxon rank-sum tests were used to analyze genome similarity. The Hasegawa-Kishino-Yano model was used for phylogenetic tree construction, with 1,000 bootstrap replicates based on the core genome-based multilocus sequence typing (cgMLST) data for *S. mutans* (Liu et al., 2020). The Phylogenetic trees were annotated using the Interactive Tree Of Life (iTOL ^2^; Letunic and Bork, 2021).

### Core gene and differentially distributed gene analyses

Whole genome sequencing and assembly were performed as detailed in our previous study (Liu et al., 2020). The protein sequences of multiple samples were clustered using Cd-hit 4.6.1. Genes were identified with default minimum amino acid sequence identity and coverage thresholds (50 and 70%, respectively) compared to the *S. mutans* UA159 reference genome (Kovac et al., 2016). Genes detected in all 80 of these samples were identified as core genes, with all other genes being denoted as non-core genes. Differentially distributed genes (DDGs) are defined as non-core genes with different distribution frequencies (*p* < 0.05) across antagonistic and non-antagonistic groups.

### Pathway annotation

KEGG pathways and GO terms associated with core and non-core genes were identified using the Database for Annotation, Visualization and Integrated Discovery (DAVID) gene annotation tool^3^ (Huang et al., 2007; Huang da et al., 2009).

### Secondary metabolite biosynthetic gene clusters analysis

AntiSMASH is a widely used tool for detecting and characterizing biosynthetic gene clusters in microorganisms (Blin et al., 2021). We used the antiSMASH bacterial version^4^ to identify secondary metabolite biosynthetic gene clusters in *S. mutans*.

### RNA-sequencing

Bacteria were cultured overnight in BHI broth, after which total bacterial RNA was extracted with the RNeasy Mini Kit (Qiagen, German) following the treatment of these bacterial suspensions with lysozyme for 40 min at 37°C. Random hexamer primers were used for reverse transcription-mediated first-strain cDNA synthesis, after which second-strand cDNA synthesis was performed. An A-Tailing Mix and RNA Index Adapters were then incubated with samples to facilitate end repair and sample indexing. The cDNA fragments were then amplified *via* PCR and purified using Ampure XP Beads. The resultant double-stranded PCR products were then denatured *via* heating and circularized with the splint oligo sequence to produce the final single-stranded circular DNA (ssCirDNA) library, which was amplified using phi29 to yield a DNA nanoball (DNB) with >200 copies per molecule. DNBs were then loaded into a patterned nanoarray, and paired-end 100 base reads were generated using the BGIseq500 platform (BGI Genomics, Shenzhen, China). Sample preparation was performed in triplicate.

### Differential gene expression analysis

Sequencing data were filtered using SOAPnuke (v1.5.2), removing those reads that contained adapter sequences, exhibited a low-quality base ratio (base quality ≤5) of >20%, and showed an unknown base (‘N’ base) ratio > 5% (Li et al., 2008). The remaining clean reads were stored in the FASTQ format. Clean reads were aligned to the *S. mutans* UA159 reference genome using Bowtie2 2.2.5 (Langmead and Salzberg, 2012), then RSEM 1.2.12 was used to calculate gene expression levels (Li and Dewey, 2011). Differential expression analyses were conducted with DESeq2 1.4.5, using the following criteria for differential expression: adjusted value of *p* (*p*_adj_) < 0.05 and fold change ≥2 or ≤ 0.5 (Love et al., 2014).

### Comprehensive analysis of genes overlapping in core genes, DDG, antiSMASH, and RNA-seq results

Genes shared among different sets of results were compared using the interactive Venny 2.1 Venn diagram tool,^5^ with genes being compared within group A (core genes, antiSMASH, and RNA-seq) and group B (DDG, antiSMASH, and RNA-seq), separately.

### qRT-PCR Validation

A PrimeScriptTM RT Reagent Kit (Perfect Real Time; Takara) was used to prepare cDNA from RNA samples. All qRT-PCR primers were designed using Primer3web (v 4.1.0), and were based upon known sequences for all genes other than SMU_1915, SMU_1317c, and SMU_1908c (Table 2). All RT-PCR reactions were conducted in a 20 μl volume containing 10 μl of 2 × TB Green Premix Ex TaqII (Takara), 0.8 μl each of the forward and reverse primers (0.4 μm), 1 μl of cDNA (300 μg/ml), 0.4 μl of ROX Reference Dye, and 7 μl of RNase-free H_2_O. Thermocycler settings were as follows: 95°C for 30 s; 40 cycles of 95°C for 5 s; and 58°C for 34 s. A melt curve was generated at the end of each reaction with continuous fluorescence monitoring. Relative gene expression was assessed *via* the 2^−△△Ct^ method (Livak and Schmittgen, 2001). All qRT-PCR analyses were conducted in triplicate.

**Table 2:** Primer sequences utilized in the present study.

ID	Prime sequences
SMU_137 F	TGGTGGTATCTTTGCGGCTA
SMU_137 R	CGCTTGTAGGCTTCGTCTTC
SMU_138 F	CATCGGTCTGCCCTTGAATG
SMU_138 R	CGGAATGCGCAGAATCAAGA
SMU_139 F	CTGCTTAGTGACTGGTTGGC
SMU_139 R	CCAAAATCCAAGCACGTCCA
SMU_140 F	CTGACGACATCTCAGCCTCA
SMU_140 R	GATGCTGCCGTGCTTGATTA
SMU_141 F	GGTTATCGGCTGCTTGTCTC
SMU_141 R	ATTCCCTGTGGTGAACGAGT
SMU_150 F	GGACAGCCAAACACTTTCAAC
SMU_150 R	ATGAGTCCCCAAGTGCCTAC
SMU_151 F	TTTTGGTGGAGATAAACAAGCTG
SMU_151 R	AAAACTACAGATCCAACCGCA
SMU_518 F	GCAGCAAGGTCAGTGTTCAA
SMU_518 R	GGTAAACATGAGACCGGCAC
SMU_1915 F	GGTTCAACTGGCTTTGGTTATGC
SMU_1915 R	GCGCTTTGTGAGGAAAATCAGTC
SMU_1916 F	GCCTGAGATGGAGTTGCTTG
SMU_1916 R	GCGATTGGAGCCTTTAGTGG
SMU_1917 F	CCTGAAAAGGGCAATCACCA
SMU_1917 R	CTGATTCAATGCGGTGGGAG
SMU_1315c F	AAATACCTGCGCTCTCCCAT
SMU_1315c R	GGCTTACCCTTTATTGCAGAGG
SMU_1316c F	GCCCACAATAAGCCAAGCAA
SMU_1316c R	CCTTGCAGGCTATCTTAACATCT
SMU_1317c F	TGGAAACAGCTTCAGTGATG
SMU_1317c R	CGAGGATCTATCTTTGTATTATC
SMU_1909c F	CGAAAAGATAGTCACGGCGG
SMU_1909c R	GGTTAGGTGCTGTTCTAAGTGG
SMU_1908c F	AGTATTTAGTAGTACCTTTTGCC
SMU_1908c R	GTTTTTATCTTGCTGTATTCTC
16S rRNA F	CTGACTTGAGTGCAGAAGGGGA
16S rRNA R	CGTCAGTGACAGACCAGAGAGC

### Statistical analysis

Data were analyzed using SPSS 20.0. The relationship between the activity of the isolates to antagonize *S. gordonii* and caries status was tested by Pearson chi-squared. Differentially distributed genes were identified *via* Pearson chi-squared tests with a theoretical frequency (TF) ≥ 5 (≤ 20% cell), while Fisher’s exact test was used in other cases. The Shapiro–Wilk test was used to establish whether qRT-PCR data were parametric, with *t*-tests and Mann–Whitney U-tests, respectively, being used to evaluate differentially expressed genes in parametric and nonparametric datasets. Correlations were analyzed using Spearman’s rank correlation coefficients. *p* < 0.05 was the threshold of significance.

## Results

### Estimation of average nucleotide identity and genetic diversity

**Figure 3:**
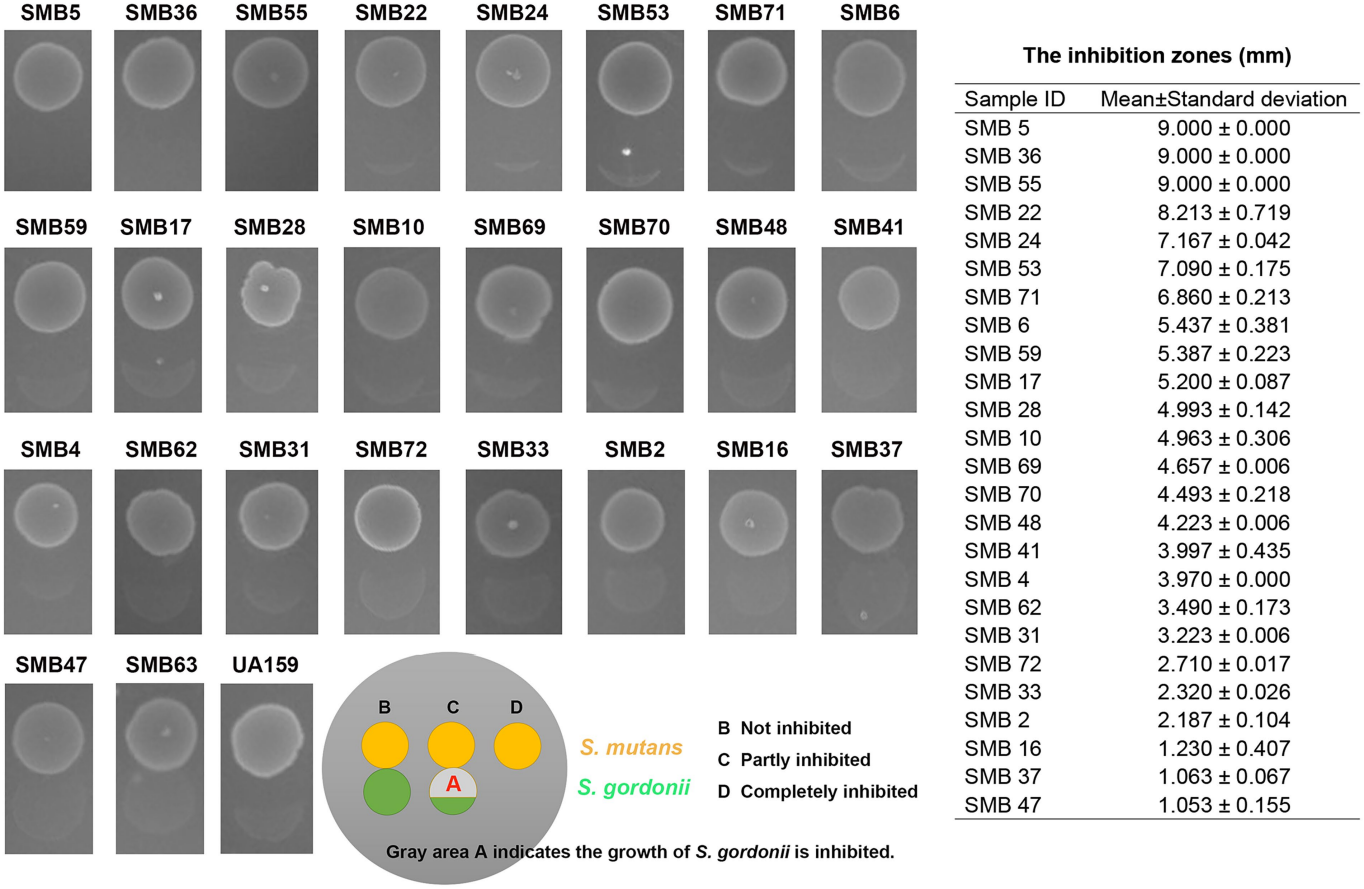
Bacteriocin assay. We used bacteriocin assay to detect the activity of *Streptococcus mutans* against *Streptococcus gordonii* in all samples. Here, we showed the results of isolates in antagonistic group, and displayed the result of SMB63 in the non-antagonistic group. SMB55 and SMB63 were the samples used in the RNA sequencing. The strain UA159 has been chosen as a positive control. The size of inhibition zones of the 25 *S. mutans* clinical isolates in the antagonistic group were also measured.

For the 80 clinical *S. mutans* isolates included in the present study, the minimum ANI value for these isolates in a BLAST comparison with *S. mutans* UA159 was 98.76%. In contrast, the maximum ANI value for 40 other species, including 38 Streptococci, *F. periodonticum*, and *N. sicca* was 78.78% compared to *S. mutans* UA159. Of these 80 clinical isolates, 25 were capable of inhibiting *S. gordonii* growth (antagonistic group) while the remaining 55 were not (non-antagonistic group; Figure 3).

**Figure 4:**
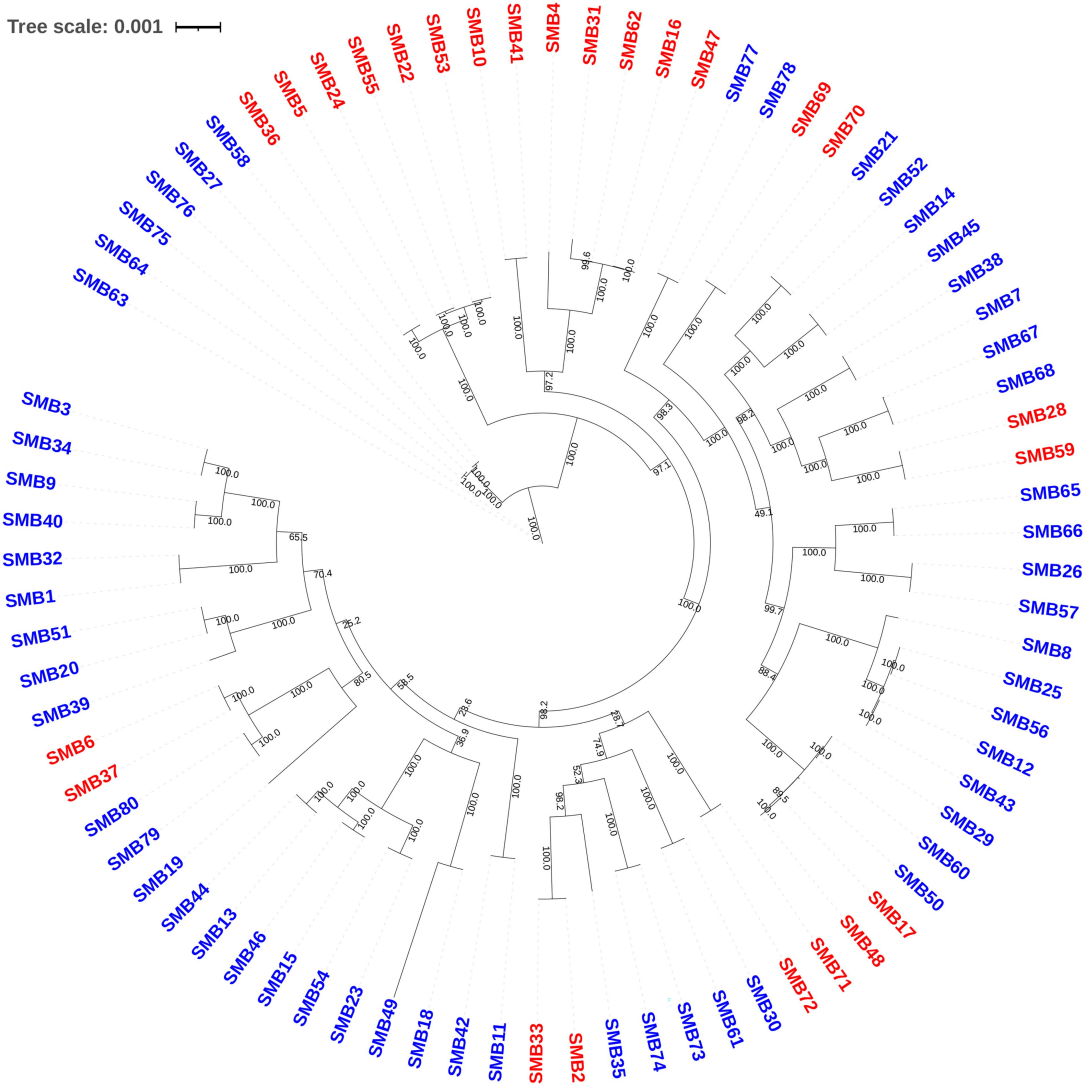
Phylogenetic tree analysis of *S. mutans* isolates from the mutacin IV and mutacin IV-free groups. A phylogenetic tree was constructed according to the cgMLST scheme and assembled using the HKY model. Numbers on lines denote bootstrap values determined for 1,000 replicates. Isolates in the mutacin IV group are marked with red color.

**Figure 5:**
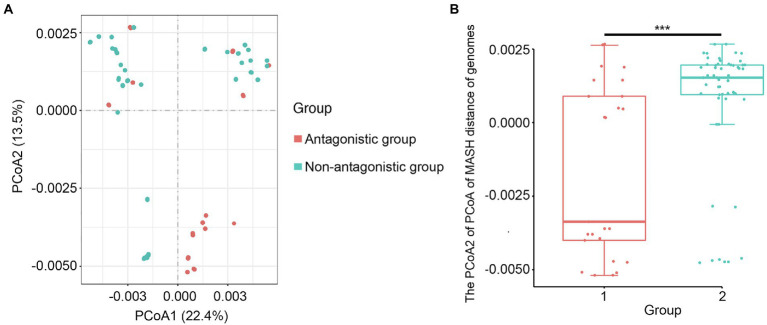
Principal Coordinate Analysis of *S. mutans* strains in the antagonistic and non-antagonistic groups. **(A)** Principal Coordinate Analysis (PCoA) of the genomes of 80 *S. mutans* clinical isolates from the antagonistic group and non-antagonistic group. **(B)** Principal component axis 2 of PCoA in **(A)** versus the group. ^***^ represents a value of *p* < 0.001.

In the antagonistic group, 21 samples were from the caries population, and 4 samples were from the caries-free population. In the non-antagonistic group, 41 samples were from the caries population and 14 samples were from the caries-free population. The sample metadata including antagonist and caries classification are listed in Supplementary Table 1. The ability of *S. mutans* to inhibit *S. gordonii* growth in the 80 isolates has no significant relation to dental caries (*p* = 0.348). Therefore, the presence of dental caries does not affect the outcome. Phylogenetic tree analysis showed that although not all the isolates in the antagonistic group cluster together, the isolates between the two groups still showed different cluster distribution. Not all the isolates in the antagonistic or the antagonistic group clustered together, this may be due to differences in other virulence phenotypes of these isolates (Figure 4). In order to determine whether there are differences between the two groups, we further used a Principal Coordinate Analysis (PCoA). The Wilcoxon rank-sum test of variance confirmed a significant association between genome similarity and antagonism to *S. gordonii* (*p* < 0.001; Figure 5). Dissimilarity values derived from the PCoA revealed that the genomes of these isolates clustered following their effectiveness against *S. gordonii*.

### Core genes and associated pathways

In total, 1893 genes were mapped to the *S. mutans* UA159 genome in these 80 isolates, 1,432 of which were shared by all isolated strains. The reference strain was *S. mutans* UA159 (ATCC 700610). Gene ontology (GO) term and Kyoto Encyclopedia of Genes and Genomes (KEGG) pathway enrichment analyses were conducted using the Database for Annotation, Visualization and Integrated Discovery (DAVID) platform as a preliminary approach to exploring the functional roles of these core genes. KEGG analysis showed that 1,005 of these genes were enriched in 12 KEGG pathways, including the metabolic, biosynthesis of secondary metabolites, ABC transporters, biosynthesis of antibiotics, biosynthesis of amino acids, microbial metabolism in diverse environments, pyrimidine metabolism, purine metabolism, peptidoglycan biosynthesis, pyruvate metabolism, 2-Oxocarboxylic acid metabolism, and carbon metabolism pathways in *S. mutans* (Figure 1A). Moreover, 806 of these genes were associated with 10 GO terms including cell division, translation, plasma membrane, cytoplasm, ATP binding, magnesium ion binding, RNA binding, rRNA binding, transporter activity, and GTP binding functions (Figure 1B).

### Non-core genes and associated pathways

Only 21 of the remaining 461 non-core genes were found to be related to 4 pathways, including the phosphotransferase system (PTS), fructose and mannose metabolism, folate biosynthesis, and glycolysis (or gluconeogenesis) pathways according to KEGG analysis (Figure 2A). Moreover, 73, 116, and 86 genes were associated with 10, biological processes, 1 cellular component, and 7 molecular function GO terms, respectively (Figure 2B).

### Differentially distributed genes

There were 94 differentially distributed genes (DDGs) between the antagonistic and non-antagonistic groups, which include 33 and 61 genes that were positively and negatively associated with the activity of *S. mutans* against *S. gordonii*, respectively (*p* < 0.05). The data including the distribution and comparison of core genes and non-core genes in 80 samples are listed in Supplementary Table 2.

### Secondary metabolite biosynthetic gene clusters

**Figure 6:**
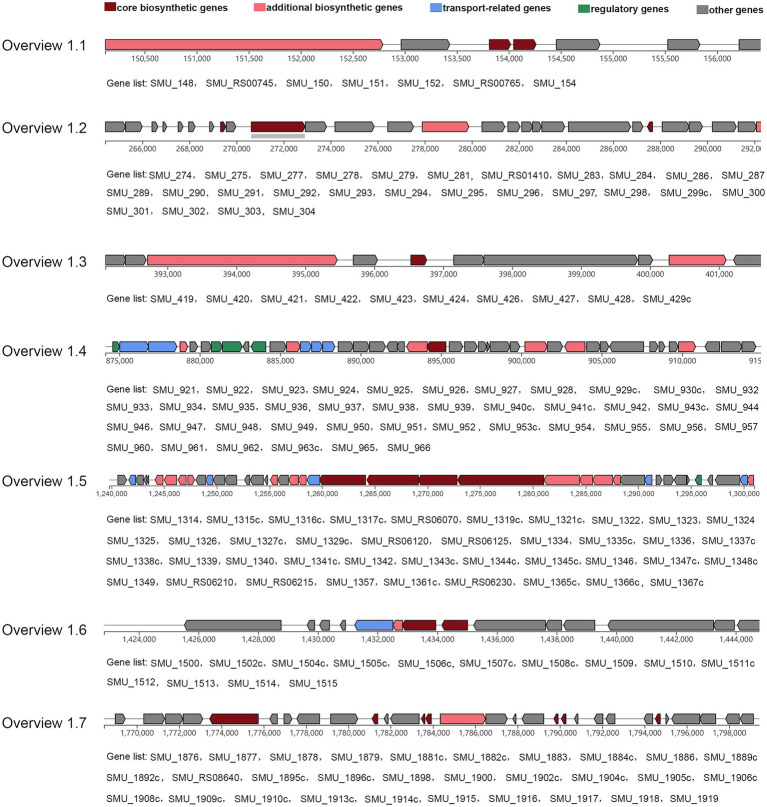
Seven clusters predicted by antiSMASH. The gene cluster contains five types of genes, including core biosynthetic genes, additional biosynthetic genes, transport-related genes, regulatory genes, and other genes. All genes involved in the corresponding cluster are attached.

An antiSMASH analysis revealed 7 secondary metabolite biosynthetic gene clusters in *S. mutans* consisting of 169 genes, including core biosynthetic genes, additional biosynthetic genes, transport-related genes, regulatory genes, and other genes (Figure 6).

### Transcriptomic analyses of differentially expressed genes and associated pathway

As a prominent isolate for the antagonistic group, SMB55 wasselected randomly from three isolates (SMB5, SMB36, and SMB55) that could entirely inhibit the growth of *S. gordonii*. SMB63 was chosen randomly from 55 non-antagonistic samples to serve as the non-antagonistic group’s representative strain. Comparing the transcriptomes of isolates that fully inhibit *S. gordonii* growth and isolates that do not inhibit *S. gordonii* growth will allow us to better screen the target genes involved in regulating *S. mutans* anti-*S. gordonii* activity. Consequently, RNA sequencing (RNA-seq) was conducted to explore differential gene expression between the antagonistic and non-antagonistic groups using the underlined isolates with three repetitions. In total, 54 genes were differentially expressed between these groups (*p*_adj_ < 0.05 and Fold-change ≥2.00 or ≤ 0.5 with a minimum of 100 average reads in either group), of which 11 (Table 3) and 43 (Table 4) were, respectively, upregulated and downregulated in the antagonistic group relative to the non-antagonistic group. The expression levels of the detected genes (with a minimum of 100 average reads in either group) are listed in Supplementary Table 3. According to KEGG pathway analysis, these differentially expressed genes are primarily involved in the biosynthesis of secondary metabolites (Supplementary Table 4).

**Table 3:** Upregulated genes determined using RNA-seq analysis.

Gene ID	Production	Mean FPKM		*p*_adj_
		Antagonistic group	Non-antagonistic group	
SMU_53	hypothetical protein	227	0	<0.001
SMU_RS00245	hypothetical protein	92	0	<0.001
SMU_52	hypothetical protein	12	0	<0.001
SMU_961	hypothetical protein	181	30	<0.001
SMU_957	50S ribosomal protein L10	1,402	242	<0.001
SMU_962	dehydrogenase	270	53	<0.001
SMU_39	hypothetical protein	133	38	0.002
SMU_1053	redox-sensing transcriptional repressor Rex	117	20	0.003
SMU_1517	response regulator CovR	140	46	0.009
SMU_1013c	Mg2+/citrate transporter	219	54	0.009
SMU_1054	glutamine amidotransferase	203	65	0.01

**Table 4:** Downregulated genes determined using RNA-seq analysis.

Gene ID	Production	Mean FPKM		*p*_adj_
		Antagonistic group	Non-antagonistic group	
SMU_1571	MsmK-like ABC transporter ATP-binding protein	490	1,037	<0.001
SMU_138	malate permease	356	3,084	<0.001
SMU_139	oxalate decarboxylase	278	2,238	<0.001
SMU_140	glutathione reductase	360	2,613	<0.001
SMU_1569	maltose ABC transporter permease	266	571	<0.001
SMU_141	hypothetical protein	174	1,515	<0.001
SMU_179	hypothetical protein	1847	5,192	<0.001
SMU_137	malate dehydrogenase	214	2019	<0.001
SMU_184	ABC transporter metal binding lipoprotein	412	658	<0.001
SMU_48	phosphoribosylamine–glycine ligase	477	973	<0.001
SMU_1570	maltose ABC transporter permease	456	966	<0.001
SMU_1232c	hypothetical protein	479	1,483	<0.001
SMU_270	PTS system ascorbate-specific transporter subunit IIC	417	680	<0.001
SMU_148	bifunctional acetaldehyde-CoA/alcohol dehydrogenase	3,287	8,200	<0.001
SMU_298	hypothetical protein	252	429	<0.001
SMU_503c	hypothetical protein	74	207	<0.001
SMU_180	oxidoreductase	3,461	7,083	<0.001
SMU_609	40K cell wall protein	2,290	5,527	<0.001
SMU_51	5-(carboxyamino)imidazole ribonucleotide synthase	900	1,509	<0.001
SMU_290	L-ascorbate 6-phosphate lactonase	603	5,517	0.001
SMU_308	sorbitol-6-phosphate 2-dehydrogenase	319	677	0.001
SMU_50	5-(carboxyamino)imidazole ribonucleotide mutase	687	1,354	0.001
SMU_309	regulator of sorbitol operon	533	1,027	0.001
SMU_118c	esterase	380	712	0.002
SMU_32	Amidophosphoribosyltransferase	453	834	0.003
SMU_662	hypothetical protein	94	336	0.003
SMU_37	bifunctional phosphoribosylaminoimidazolecarboxamide formyltransferase/IMP cyclohydrolase	793	1,646	0.004
SMU_1613c	dephospho-CoA kinase	289	540	0.005
SMU_1602	NAD(P)H-flavin oxidoreductase	905	1,519	0.005
SMU_1862	hypothetical protein	1,537	3,371	0.005
SMU_2127	succinate semialdehyde dehydrogenase	1,354	2,797	0.006
SMU_1539	glycogen branching protein	734	1,270	0.007
SMU_448	hypothetical protein	177	321	0.009
SMU_RS05810	phosphoribosyl-ATP diphosphatase	32	117	0.009
SMU_35	phosphoribosylglycinamide formyltransferase	477	800	0.009
SMU_435	N-acetylglucosamine-6-phosphate deacetylase	133	229	0.017
SMU_1267c	hypothetical protein	284	697	0.018
SMU_1975c	hypothetical protein	72	126	0.018
SMU_1889c	hypothetical protein	22	412	0.019
SMU_34	phosphoribosylaminoimidazole synthetase	269	469	0.019
SMU_844	hypothetical protein	126	244	0.023
SMU_291	Transketolase	1,201	2,490	0.025
SMU_181	mevalonate kinase	126	202	0.021

### Comprehensive analysis of the common genes across the core gene/DDG, antiSMASH, RNA-seq, and KEGG pathway analyses

**Figure 7:**
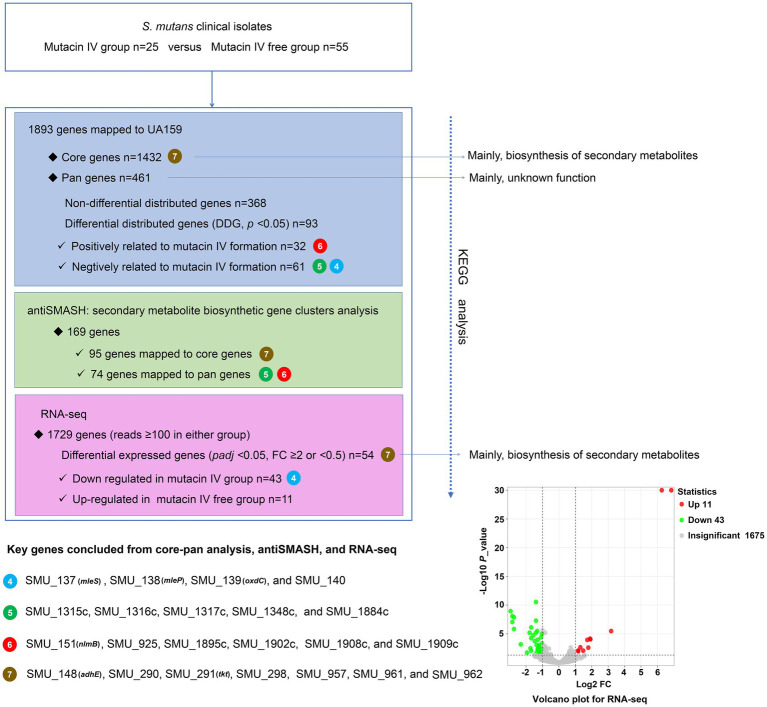
Overview of genomic comparison, antiSMASH, RNA-seq, and KEGG pathway analyses. The circle represents the shared genes discovered through various investigations, and the number within the circle shows the number of shared genes.

Of the 169 genes obtained through antiSMASH analyses, 95 belonged to the core gene set identified above. Of the remaining 74 genes, 5 (SMU_1315c, SMU_1316c, SMU_1317c, SMU_1348c, and SMU_1884c) were negatively correlated with the activity of *S. mutans* against *S. gordonii*, while 6 (SMU_151, SMU_925, SMU_1895c, SMU_1902c, SMU_1908c, and SMU_1909c) were positively correlated with this effect. RNA-seq analyses revealed 4 downregulated genes were mapped to the DDGs (SMU_137-SMU_140). In total, 7 genes were shared across the core gene, antiSMASH, and RNA-seq analyses. When RNA-seq and DDG results were correlated, we found that SMU_137-SMU_140 were downregulated in the antagonistic group in RNA-seq results and negatively associated with the activity of *S. mutans* against *S. gordonii*. Figure 7 illustrates the complex relationship described above.

### Expression levels validation of mRNAs based on qRT-PCR

**Figure 8:**
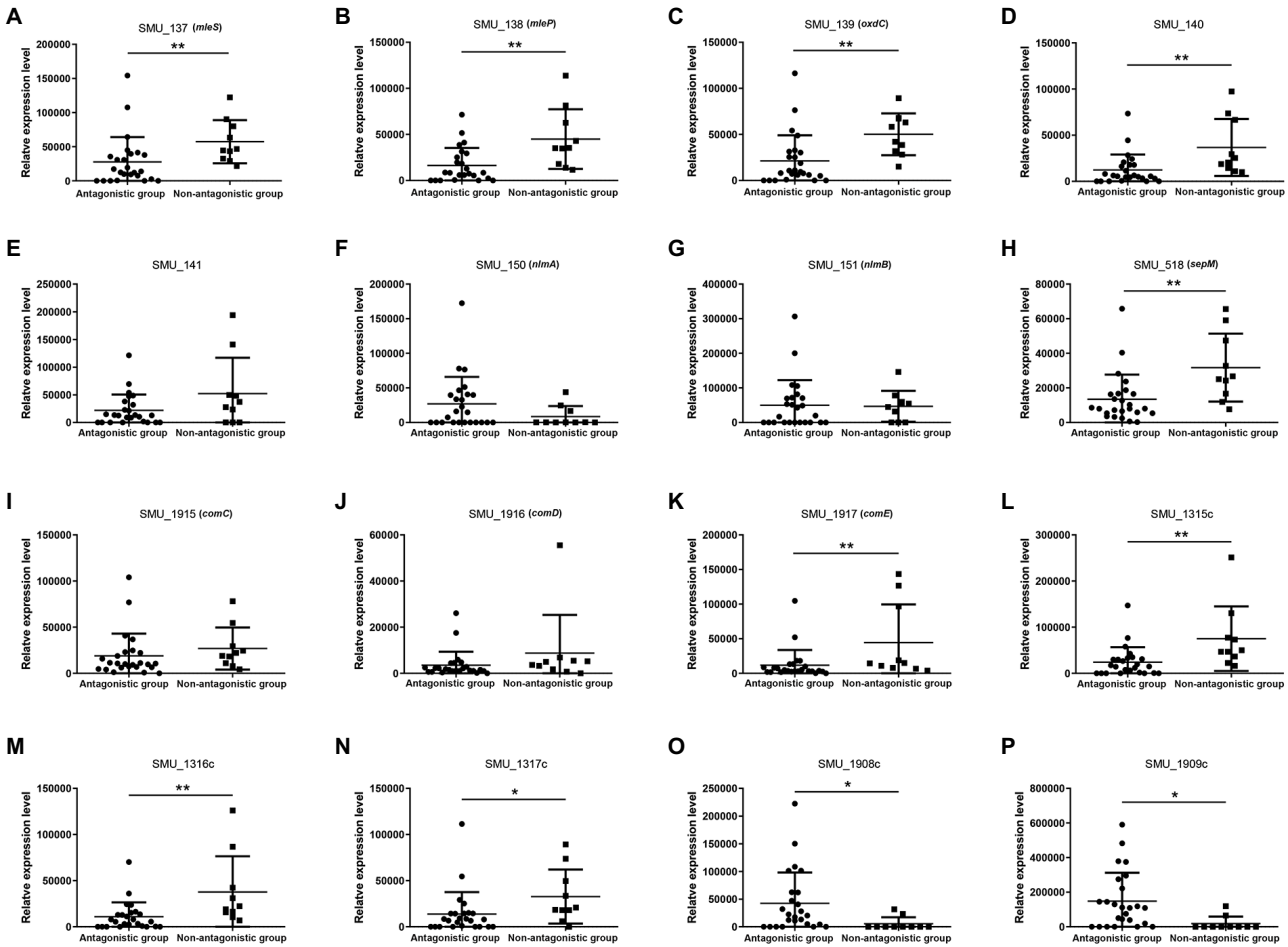
Gene expression levels for 16 selected genes (SMU_137–141, SMU_150, SMU_151, SMU_518, SMU_1915–1917, SMU_1315c-SMU_1317c, SMU_1908c, and SMU_1909c) as measured by qRT-PCR in the antagonistic group and non-antagonistic group. ^*^ represents value of *p* < 0.05, ^**^ represents value of *p* < 0.01. The expression level of SMU_137 in SMB16 was defined as 1. The experiment on 35 strains was conducted in three technical replicates. The CT values of the qRT-PCR is listed in Supplementary Table 5.

**Figure 9:**
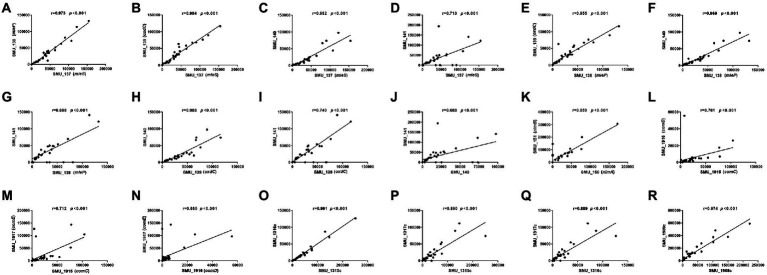
Spearman-rank correlation coefficient analysis of adjacent genes.

We chose qRT-PCR to verify the results of RNA-seq, including more clinical samples, because the obtained results from these two samples (SMB55 and SMB63) could not adequately represent the transcriptome comparison results of the two groups (the antagonistic and the non-antagonistic groups). Overall, sixteen genes (SMU_137, SMU_138, SMU_139, SMU_140, SMU_141, SMU_150, SMU_151, SMU_518, SMU_1915, SMU_1916, SMU_1917, SMU_1315c, SMU_1316c, SMU_1317c, SMU_1908c, SMU_1909c) were selected for qRT-PCR-based validation performed using 35 clinical isolates, including 25 from the antagonistic group and 10 that were randomly selected from the non-antagonistic group. SMU_137, SMU_138, SMU_139, SMU_140, SMU_1315c, SMU_1316c, and SMU_1317c were downregulated in the antagonistic group, whereas SMU_1908c and SMU_1909c were upregulated in this group (Figure 8). The average SMU_150 and SMU_151 expression levels were higher in the antagonistic group than in the non-antagonistic group, but the difference was insignificant. Adjacent genes exhibited closely related expression levels (Figure 9). For example, SMU_137, SMU_138, SMU_139, and SMU_140 exhibited strongly positively correlated expression patterns, with correlation coefficient values greater than 0.9. Similarly, SMU_1908c and SMU_1909c were strongly positively correlated (*r* = 0.974), as were SMU_1315c and SMU_1316c (*r* = 0.991).

## Discussion

The current study investigated potential key genes involved in regulating the activity of *S. mutans* that inhibit the growth of *S. gordonii* using 80 *S. mutans* clinical isolates isolated from supragingival dental plaques of children with early childhood caries and caries-free, using a combination of comparative genome analysis, RNA-seq, antiSMASH, and qRT-PCR between antagonistic and non-antagonistic groups.

PCoA analysis of the antagonistic and non-antagonistic groups revealed significant variations in their genetic features. By comparing the genomes of samples from the two groups, we may identify critical target genes for *S. mutans* to inhibit *S. gordonii* growth. There were 1,432 core genes and 94 DDGs discovered in this study. The number of core genes identified here is approximately 30% higher than the number of core genes (1083) identified by Meng et al. (2017). The following two factors could explain this: (1) 80 *S. mutans* clinical isolates used in this study were collected from children living in Bengbu, China, while the 183 *S. mutans* isolates used in that study included 11 genomes assembled by authors and 172 previously published genomes. These isolates were obtained from Brazil, Iceland, South Africa, Turkey, the USA, and the rest. Our earlier investigation demonstrated that the significant allele heterogeneity of *S. mutans* among different countries is apparent (Liu et al., 2020). Because these strains originate from diverse regions, the isolation difference may be substantial, and their core gene count would be much lower than in this study and (2) The serotype c was identified in 80 *S. mutans* clinical isolates used in this study, but the serotypes of the isolates in that study were diverse. For example, *S. mutans* LJ23 and *S. mutans* UA159 are serotype k and serotype c, respectively, which may also affect the acquisition of core genes. According to KEGG pathway analysis, core genes are primarily involved in thesecondary metabolite biosynthesis, and the function of most DDGs isunidentified and needs to be explored. We believe DDGs are essential in the competition between *S. mutans* and *S. gordonii*.

Bacteriocins produced by lactic acid bacteria (LAB) are primarily active against other gram-positive bacteria that are closely related (Zheng and Sonomoto, 2018). *Streptococcus mutans*, a lactic acid bacterium, can produce mutacin IV against various streptococcal species growth including *S. gordonii* (Kreth et al., 2008). The nlmT (SMU_286), nlmE (SMU_287), sepM (SMU_518), comC (SMU_1915), comD (SMU_1916), and comE (SMU_1917) genes have previously been reported to be positive regulators of mutacin IV production, with nlmA (SMU_150) and nlmB (SMU_151) encoding mutacin IV (Hale et al., 2005; van der Ploeg, 2005; Hossain and Biswas, 2012a). SMU_152, topologically linked to nlmAB operon, acts as an immunity protein giving protection against mutacin IV in *S. gordonii* (Hossain and Biswas, 2012b). The present study identified nlmT, nlmE, sepM, comC, and comE as core genes. All but one isolate from the non-antagonistic group expressed comD in this study. The overall distribution frequency of nlmA and SMU_152 across both groups was 56%. The distribution frequency of nlmB in the antagonistic group was significantly higher than that in the non-antagonistic group (56% vs. 20%). Additionally, except for sepM, there was no statistically significant variation in the expression levels of nlmT, nlmE, nlmA, nlmB, comC, comD, and comE between the two groups according to RNA-seq analysis. These results suggest that, in addition to the mutacin IV -related genes, other key genes are involved in *S. mutans* anti-*S. gordonii* antagonism. These findings highlight the significance of our current research, which is focused on identifying candidate targets against *S. gordonii* in *S. mutans* clinical isolates.

The antiSMASH is an effective tool used extensively to screen bacterial biosynthetic gene clusters (Chakraborty, 2022). A previous study showed that nlmTE encoding of the ABC transport system is required to export nonlantibiotic mutacins in *S. mutans* (Hale et al., 2005). The antiSMASH also predicted 11 genes (SMU_922, SMU_923, SMU_934, SMU_935, SMU_936, SMU_1315c, SMU_1325, SMU_1338c, SMU_1348c, SMU_1366c, SMU_1506c) belonged to transport-related genes. To fully explore genes that may play a role in regulating the activity of *S. mutans* against *S. gordonii*, we combined results from core gene, DDG, RNA-seq, and antiSMASH analyses to select 16 genes for qRT-PCR-based validation in 35 *S. mutans* isolates. These validation results revealed that SMU_137 (malate dehydrogenase, mleS), SMU_138 (malate permease, mleP), SMU_139 (oxalate decarboxylase, oxdC), and SMU_140 were downregulated in isolates from the antagonistic group, consistent with RNA-seq results. Per the DDG analysis, SMU_1315c, SMU_1316c, and SMU_1317c were negatively associated with the activity of *S. mutans* against *S. gordonii*, whereas SMU_151, SMU_1908c, and SMU_1909c were positively related to this function. Consistently, our qRT-PCR results indicated that SMU_1315c, SMU_1316c, and SMU_1317c were downregulated in the antagonistic group, while SMU_1908c and SMU_1909c were upregulated in this group. Malolactic fermentation (MLF) has been identified as a primary mechanism whereby *S. mutans* and other oral streptococci induce alkalinity (Sheng and Marquis, 2007), and this pathway plays a central role in protecting *S. mutans* against acidic damage, oxidative stress, and starvation (Sheng and Marquis, 2007). MLF is important in protecting *S. mutans* against acid damage and oxidative and starvation damage (Sheng et al., 2010). SMU_137, SMU_138, and SMU_139 are the main genes associated with MLF in *S. mutans* (Lemme et al., 2010). However, the relationship between these genes and the activity of *S. mutans* against *S. gordonii* remains unknown. Our data suggest that SMU_137–140, SMU_1315c-1317c, and SMU_1908c-1909c may play key roles in regulating this function, and SMU_1315c may be the transport-related gene. As we further found that adjacent genes exhibit closely related patterns of expression, these data maysuggest synergistic roles for these gene clusters. The cooperative regulation relationship between genes in gene clusters has also been reported before. SMU_150 and SMU_151, for instance, are mutacin IV co-coding genes that shared a single operon (Qi et al., 2001). *S. mutans* produces mutacin IV, a bacteriocin that inhibits *S. gordonii* growth. SMU_150 and SMU_151 work together to control mutacin IV production. SMU_1915-SMU_1917, particularly SMU_1916 and SMU_1917, are positively correlated with mutacin IV production (Hossain and Biswas, 2012a). Phosphorylation of SMU_1916 causes phosphorylation of SMU_1917, which then interacts directly with the promoters of SMU_ 150 and SMU_ 151, promoting mutacin IV synthesis. Figure 9 shows the expression connection of these neighboring. Nevertheless, the mechanism by which these target gene clusters govern *S. mutans*and inhibit *S. gordonii* has not been revealed. Future investigation is required, including gene knockdown and post-transcriptional regulation.

## Conclusion

*Streptococcus mutans* is an important cariogenic bacterium that causes dental caries, whereas *S. gordonii* is a non-cariogenic pioneer bacterium that colonizes the tooth surface and inhibits the growth of *S. mutans*. Identifying *S. mutans*’s essential genes against *S. gordonii* has substantial therapeutic implications for preventing and treating of dental caries. The findings of our study demonstrate that genome comparison help determine potential target genes in regulating the interaction between *S. mutans* and *S. gordonii*; SMU_137–140, SMU_1315c-1317c, and SMU_1908c-1909c were identified as 3 potential candidate gene clusters controlling this activity, with SMU_1315c possibly being a transport-related gene; adjacent genes in the gene cluster may synergistically regulate the activity of *S. mutans* against *S. gordonii* growth. These findings point toward a new direction for studying *S. mutans*’ cariogenic mechanism through the prism of bacterial interaction.

## Data availability statement

The datasets presented in this study can be found in online repositories. The names of the repository/repositories and accession number(s) can be found at: https://www.ncbi.nlm.nih.gov/, PRJNA804356.

## Ethics statement

The studies involving human participants were reviewed and approved by The First Affiliated Hospital of Bengbu Medical College provided ethical approval ([2017] KY011) for the present study. Written informed consent to participate in this study was provided by the participants’ legal guardian/next of kin.

## Author contributions

SL and KZ contributed to conception and design of the study. SL, YS, YL, and LX analyzed the database. QZ, QW, and GZ assisted in data analysis. SL and FH performed the statistical analysis. SL wrote the first draft of the manuscript. YS wrote sections of the manuscript. All authors contributed to the article and approved the submitted version.

## Funding

This work was supported by the National Natural Science Foundation of China (Grant no. 32000386), the First Affiliated Hospital of Bengbu Medical College Science Fund for Outstanding Young Scholars (Grant no. 2019byyfyyq07), and the University Synergy Innovation Program of Anhui Province, China (Grant no. GXXT-2021-056).

## Conflict of interest

The authors declare that the research was conducted in the absence of any commercial or financial relationships that could be construed as a potential conflict of interest.

## Publisher’s note

All claims expressed in this article are solely those of the authors and do not necessarily represent those of their affiliated organizations, or those of the publisher, the editors and the reviewers. Any product that may be evaluated in this article, or claim that may be made by its manufacturer, is not guaranteed or endorsed by the publisher.

## Footnotes

^1^ https://github.com/marbl/Mash

^2^ https://itol.embl.de/

^3^ https://david.ncifcrf.gov/

^4^ https://antismash.secondarymetabolites.org

^5^ https://bioinfogp.cnb.csic.es/tools/venny/index.html
